# Green Chemically Synthesized Iron Oxide Nanoparticles–Chitosan Coatings for Enhancing Strawberry Shelf-Life

**DOI:** 10.3390/polym16233239

**Published:** 2024-11-22

**Authors:** Ayesha Sani, Dilawar Hassan, Ghulam Qadir Chanihoon, Dulce Viridiana Melo Máximo, Elvia Patricia Sánchez-Rodríguez

**Affiliations:** 1 Tecnologico de Monterrey, School of Engineering and Sciences, Atizapan de Zaragoza 52926, Estado de Mexico, Mexicovirimelo@tec.mx (D.V.M.M.); elvia.sanchez@tec.mx (E.P.S.-R.); 2 National Centre of Excellence in Analytical Chemistry, University of Sindh Jamshoro, Jamshoro 76080, Sindh, Pakistan; chemistqadir@gmail.com

**Keywords:** natural extracts, biodegradable, chitosan, food preservation, strawberries, composite coatings, nanoparticles

## Abstract

To enhance the preservation of strawberries, a novel coating formulation was developed using chitosan (CH) and iron oxide (IO) nanoparticles (NPs) supplemented with ginger and garlic extracts and combined with varying concentrations of 1%, 2%, and 3% Fe_3_O_4_ NPs. The results of XRD revealed an average crystalline size of 48.1 nm for Fe_3_O_4_ NPs. SEM images identified Fe_3_O_4_ NPs as bright spots on the surface of the fruit, while FTIR confirmed their presence by detecting specific functional groups. Additional SEM analysis revealed clear visibility of CH coatings on the strawberries. Both uncoated and coated strawberry samples were stored at room temperature (27 °C), and quality parameters were systematically assessed, including weight loss, firmness, pH, titratable acidity (TA), total soluble solids (TSSs), ascorbic acid content, antioxidant activity, total reducing sugars (TRSs), total phenolic compounds (TPCs), and infection rates. The obtained weight loss was 21.6% and 6% for 1.5% CH and 3% IO with 1.5% CH, whereas the obtained infection percentage was 19.65% and 13.68% for 1.5% CH and 3% IO with 1.5% CH. As strawberries are citric fruit, 3% IO with 1.5% CH contains 55.81 mg/100 g ascorbic acid. The antioxidant activity for 1.5% CH coated was around 73.89%, whereas 3% IO with 1.5% CH showed 82.89%. The studies revealed that coated samples showed better results, whereas CH that incorporates Fe_3_O_4_ NP coatings appears very promising for extending the shelf life of strawberries, preserving their quality and nutritional value during storage and transportation.

## 1. Introduction

The food industry is progressively becoming characterized by a dedication to maintaining food quality and preventing microbial growth that leads to spoilage [1]. Traditionally, this has involved the use of chemical additives and high-temperature treatments to resist bacterial and fungal growth. However, these practices adversely affect vitamins and minerals in food [2,3]. As consumer awareness of the potential side effects of chemicals grows, there is an increasing demand for food authorities to ensure the delivery of safe and healthy food. This shift has led to a reduction in the reliance on antibiotics and synthetic additives that have been commonly used to protect against pathogens that can cause food spoilage.

Nowadays, consumer habits have been changing in favor of chemically untreated food or food with low chemical residue while maintaining quality standards. Food authorities worldwide are directing more of their attention toward various aspects of food security, including quality, safety, and preservation. This has led to an inquiry regarding natural agents constituting plant extracts or byproducts, enzymes, and microorganisms, such as fungi and bacteria [4,5]. Biopreservation methods are emerging as increasingly popular and relatively safer alternatives to chemical preservatives. Biopreservation not only enhances the shelf life of foods but also preserved their nutritional values and combats pathogenic microorganisms. These methods also promise to enhance food quality through their antimicrobial, antioxidant, and anti-inflammatory properties [3,6]. Therefore, the food authorities must ensure the production of safe and healthy food for consumers, as people are conscious about the side effects of chemicals. Food providers add antibiotics and synthetic additives to preserve food from food-spoiling pathogens. However, now, customers want high-quality food with fewer or no chemicals and it is favorable to use natural compounds [4,7].

Research in the field of active food packaging, i.e., packages containing active additives with a key role in food preservation, is mapping a path to boost the safety, quality, and shelf-life of packaged foods by reducing food spoilage, waste, and recalls, as well as foodborne illness outbreaks [8]. Nonetheless, a significant portion of these active packaging solutions are made of non-biodegradable synthetic polymers, and their non-reusable nature gives rise to environmental challenges. Therefore, the use of eco-friendly natural polymers to develop active food packaging systems is one of the promising routes that are being pursued to reduce the environmental impact of disposable packaging materials [9]. Equally important is the use of natural compounds or extracts as bioactive additives to convey active properties to food packaging systems, along with the use of environmentally friendly film-processing methods [10]. Biopolymers are among the most investigated biodegradable materials when it comes to developing food packaging materials because of their remarkable properties, including their renewability, abundance, non-toxicity, biodegradability, biocompatibility, and functionality [11,12].

Various biopolymers, such as polysaccharides, proteins, and lipids, can be extracted from different natural sources [13]. The most used polysaccharides in the food industry are starch [14], cellulose [15], gum Arabic [16], chitin [17], chitosan [18], and alginates [19]. Polysaccharides have a strong film-forming capacity, enabling them to be used as films and coatings for packaging various food products, such as meats, fruits, and vegetables [20,21]. Among the functional components, phytochemicals derived from commonly consumed plant foods, including fruits, vegetables, grains, beans, and herbs, can serve as antibacterial agents, antioxidants, and indicators in food packaging systems [22]. These plant byproducts are renewable, non-toxic, biodegradable, biocompatible, and cost-effective raw materials for biodegradable packaging [23]. Interestingly, byproduct utilization in biodegradable packaging has the potential to reduce global warming, which usually occurs due to plastic composting and landfilling processes. Chitosan is a biofriendly material that can be safe to use for living cells. Chitosan exhibits antibacterial and antifungal properties. The reported hypothesis showed that CH-based films or coatings form a cellophane-like structure to protect food. Basically, the CH coating blocks the external microbes from reaching the food’s surface, restricts oxygen permeability and inhibits the growth of bacteria [24]. CH can also bind with different materials for functionalization, such as metal oxide nanocomposites, and creates strong hydrogen bonding which enhances its antimicrobial, antioxidant, antifungal properties [25,26,27]. There has been an increase in research exploring the benefits of plant byproducts in food packaging systems in recent years. The addition of plant extracts to biodegradable packaging films enhances their properties, including their physical, mechanical, barrier, color, functional, structural, antioxidant, and antimicrobial properties. It also modifies the properties of packaging material, which enhances its shelf life, durability and overall application [23,28].

*Allium sativum* [29], which is commonly named garlic, is a cultivated Asian plant which consists of ~65% water, ~28% carbohydrate, ~2% protein, ~1.2% amino acids, ~1.5% fibers, phenols, fatty acids and ~2.3% sulfur compounds. In ancient times, garlic was used by Indian people, Greeks, Egyptians, Chinese people, Babylonians, and Israelis as a healing medicine for various conditions such as diarrhea, fever, high cholesterol, asthma, leprosy, and more [30,31]. Garlic was also investigated for its potential to reduce cancer risk. The mortality rate is increasing as cancer cases spread day by day [32]. Research was conducted to investigate the reduction in cancer risk as a result of garlic intake. This investigation lasted for a year, and the RCT showed that the number and size of colorectal adenomas were significantly reduced in those patients who were introduced to high doses of garlic extract [33]. Garlic is a wonderful antibiotic and a strong antimicrobial agent. It exhibits broad-spectrum activity that can inhibit both Gram-positive and -negative bacteria [34]. *Zingiber officinale* [35], which is commonly named ginger, is a cultivated Asian plant that is used as a dietary supplement and for medicinal purposes for respiratory and gastrointestinal diseases. Ginger has been reported to possess various properties, such as anti-inflammatory, anticancer, antioxidant, antidiabetic, and analgesic properties [36,37]. The addition of ginger and garlic extracts causes hydrogen bonding with chitosan and improves its mechanical properties, reducing its pore size [38]. Furthermore, plant extracts are hydrophobic in nature and as such, strawberries are forced to retain their moisture, which extends their shelf-life [39]. Nanoscience deals with material in the range between 1 and 100 nm; nanoparticles of this size easily interact with and penetrate materials. In the fields of tissue engineering, regenerative medicine, and drug delivery, nanoscience is a center of research for the development of new methods. The US National Science and Technology Council has an organization named “The National Nanotechnology Initiative” that arranges various clinical setups that demonstrate NPs’ involvement in different diseases, such as cancer, kidney diseases, and respiratory diseases [40,41]. 

Metal NPs have various applications in different fields such as optics, electronics, catalysis, and the biomedical field [42]. Biofriendly materials like plants, herbs, and bacteria can be used to synthesize metallic nanoparticles which show negligible toxicity [43,44,45]. The -OH group from the biomolecules in plant extracts and chitosan help the metal oxide NPs to cause hydrogen bonding with the chitosan and biomolecules [46]. IO NPs have super magnetic properties, making them inherently biocompatible in nature due to the presence of iron. Therefore, the minimum concentration is considered biologically safe for humans compared to cobalt, silver, cadmium, etc. IO NPs are also used in MRI (Magnetic Resonance Imaging), which is used to obtain detailed internal images of the body. Another key advantage is that super magnetic IO NPs can be easily recycled or removed from the reaction medium after the completion of the reaction [47,48]. Green synthesis introduced more biofriendly IO NPs, which highlighted their antimicrobial, antioxidant, and photocatalytic properties [49]. The FDA (Federal Drug Administration) approved Endorem, which is an iron-based MRI for the liver and spleen. This consists of dextran with a core of IO NPs in the range of 50–180 nm. Clinical trials of Endorem were tested in many countries like the USA, Japan, and Europe [50,51]. Due to their magnetic nature, these NPs show a lot of agglomeration, which can be overcome by functionalizing them with other metals, drugs, organic compounds, or polymers [52]. Furthermore, the size of NPs plays a vital role in defining the barrier properties of the coatings. The larger size of the nanoparticles causes stronger bonding between the chitosan’s polymeric chains, causing them to have a compact structure and limiting their moveability, thus reducing the pore size. The reduced pore size makes the passage of water molecules more difficult, enhancing the barrier properties and reducing the water vapor permeability [53]. This study introduced a novel method for preparing a coating solution, in which analytical-grade acid was replaced by lemon juice. Additionally, ginger and garlic extracts, along with Fe_3_O_4,_ were incorporated to enhance the antimicrobial properties and extend the shelf life of strawberries at ambient temperature. Various parameters, including pH, weight loss, infection rate, and firmness, were assessed to evaluate quality and mold growth during storage.

## 2. Material and Methods

For the preparation of the coating solution, the following materials were used: strawberries (*Fragaria* × *ananassa*), garlic (*Allium Sativum*), lemon (citrus limon), and ginger (*Zingiber officinale Roscoe*), obtained from the local market in Villa de La Hacienda, Estado de Mexico, Mexico. Chitosan (C_56_H_103_N_9_O_39_), sodium hydroxide (NaOH), 3,5-diniytrosalicyclic acid (C_7_H_4_N_2_O_7_), iron (II) sulfate (FeSO_4_), and glycerol (C_3_H_8_O_3_) were all bought from Sigma Aldrich (St. Louis, MO, USA).

### 2.1. Lemon Juice and Ginger and Garlic Extraction

#### 2.1.1. Filtration of Lemon Juice

**Figure 1:**
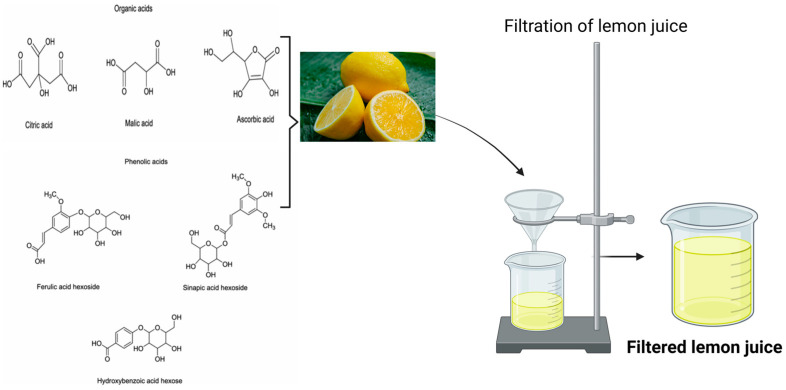
Schematic representation of filtration of lemon juice.

Lemon juice was used to prepare the coating solution. The lemons were washed properly with distilled water and cut into two pieces. To collect the lemon juice, the slices were squeezed with the help of a handheld metal squeezer prior to further processing, as detailed in Figure 1. This juice was filtered by using Whatman filter paper 40 to remove any suspended particles and stored in an airtight container for further processing. Figure 1 also showcases the possible organic compounds within the lemon juice that would help dissolve CH as well as playing their part in improving the strawberry preservation properties of the CH coating.

#### 2.1.2. Extraction of Ginger and Garlic Extract

**Figure 2:**
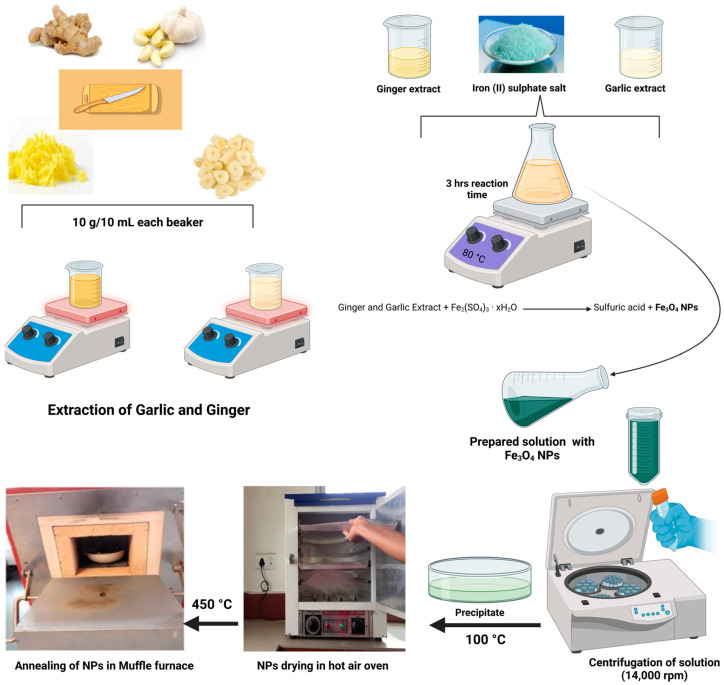
Schematic diagram of extraction of ginger and garlic extracts and synthesis of Fe_3_O_4_ NPs using a mixture of ginger and garlic extracts.

The aqueous extraction of ginger and garlic was performed using a previously reported method [54]. Briefly, 10 g of finely chopped ginger and garlic pieces were put into a 250 mL beaker. The beakers contained 100 mL of DI water. The pieces were heated at 70 °C for 3 h under constant stirring at 600 rpm to obtain various bio-compounds which were extracted from the ginger and garlic into the DI water, as shown in Figure 2. Lastly, after 3 h, the solutions were filtered using Whatman # 40 filter paper remove any remaining fibers and ginger and garlic pieces and were stored in an airtight container for further use.

### 2.2. Synthesis of Nanoparticles

NPs were synthesized using the reported method [55], with minor modifications. Briefly, 1:1 (*v*/*v*) of ginger and garlic extracts, 100 mL in total, was placed in a 250 mL beaker. The extract solutions were heated to 60 °C, followed by the introduction of 6.0 g of precursor salt (iron (III) sulfate). After the addition of salt, the temperature of the reaction solution was raised to 80 °C and the solution was left for 3 h under constant stirring. Once the reaction was complete, the solution was centrifuged at 14,000 rpm for 10 min. The precipitate appeared at the bottom of falcon tubes, the surplus liquid was removed, and DI water was added to the falcon tubes. The same centrifugation procedure was repeated thrice to eliminate any contamination. After every cycle, the water was changed to remove impurities. After three cycles, the collected precipitates were dried at 100 °C and calcinated at 450 C for 2 h to obtain assumed Fe_3_O_4_ NPs, as documented in the accompanying Figure 2.

#### Characterization Techniques of NPs

The synthesized Fe_3_O_4_ NPs were subjected to a detailed characterization that incorporated X-ray diffraction (XRD), scanning electron microscopy (SEM), and Fourier transform infrared spectroscopy (FTIR) analyses to elucidate their crystalline structure, surface morphology, and molecular bonding attributes. XRD examinations were conducted using a Bruker AXS D-8 system (GmbH, Karlsruhe, Germany), with Cu Kα. The SEM analysis was used to evaluate the surface morphology of the biosynthesized IO NPs. For the analysis, JEOL’s Field Emission Scanning Electron Microscope, model JSM-IT700 HR and JSM-6360LV (Mitaka, Tokyo, Japan), was employed. The accelerating current used for the electron gun was 10.0 kV with a working distance of WD = 41.3 mm. The FTIR analysis was conducted using Spectrum 2 FTIR by Perkin Elmer (Walthamm, MA, USA) to analyze the attachment of functional groups that could have originated from the plant extract and that contributed to the reduction in the precursor salt, with scans ranging from 4000 to 400 cm^−1^.

### 2.3. Preparation of Coating Solution

**Figure 3:**
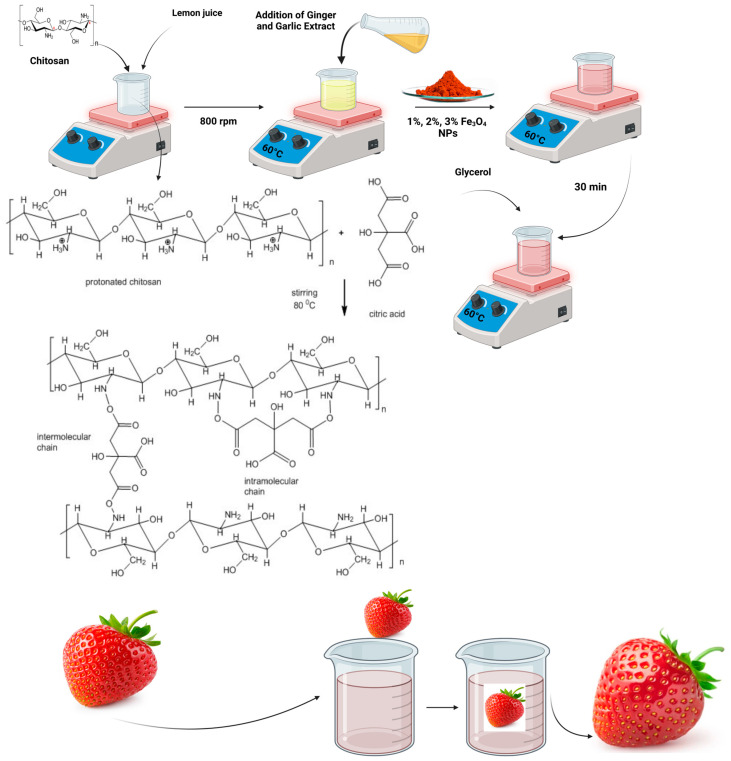
Graphical representation of the coating solution preparation and the application of prepared solution on strawberries.

Four chitosan solutions were prepared using 1.5% chitosan with different concentrations of IO NPs for the coating solution. Firstly, 1.5 g of CH was placed inside a 250 mL beaker with 50 mL of lemon juice and subjected to constant magnetic stirring at 800 rpm for 30 min. Then, 50 mL of ginger and garlic extract was poured in an equal ratio, and the solutions were left to be constantly stirred at 800 rpm for 2.5 h. After that, 1, 2, and 3% IO NPs were added into 3 different beakers with 1.5% already prepared CH solution as described in Figure 3. One beaker was left without NPs, and only 1.5% CH, to be kept as a control solution. The solution mixture with NPs was stirred for 30 min to obtain a homogenous dispersion of IO NPs and 1% weight equivalent glycerol was added. The prepared solution was then used to coat the strawberries at least thrice to ensure the complete application of the solution, as shown in Figure 3, and for other physicochemical studies.

#### Coating Solution Characterization Parameters

The rheological behavior of the coating solution was evaluated using a TA Instruments Hybrid Rheometer DHR-3 (New Castle, DE, USA), configured in a cone-and-plate setup comprising a 60 mm diameter stainless steel cone (0.9969°) and a Peltier temperature control system. Oscillatory rheology was assessed across an angular frequency spectrum of 0.1 to 100 rad/s at a constant strain of 1.5% to deduce the storage (G′) and loss (G″) moduli of the strawberry coating formulation. Furthermore, viscometry and flow curve assessments were conducted over a 0.1 to 1000 s^−1^ shear rate range. Prior to coating application, strawberries were meticulously cleansed thrice using distilled water before the application of the CH-based coating solution, which included 1%, 2%, and 3% IO NPs, as well as a control group without NPs. Moreover, SEM was utilized to analyze and compare the surface morphology of the control and NP-coated strawberries to confirm the development of the coating.

### 2.4. Characterization Parameters of the Coating Solution

Strawberries were washed properly with distilled water and dried. The strawberries were coated with 1.5% CH coating solution and 1.5% CH coating solution with 1, 2, and 3% IO NP concentrations. 

#### 2.4.1. Determination of Weight Loss and Firmness

The weight loss of the coated strawberries was determined gravimetrically. The preserved samples of strawberries were weighed before coating as *W_i_* and after storage as *W_f_* (with a time interval of 1 day). The % weight loss of coated fruit was measured by using Equation (1) [56]:

(1) $$Weight\;loss\;(\%)=\frac{\left(W_{i}-W_{f}\right)}{W_{i}}\times 100$$

The firmness of the fruit was measured by the puncture test using a texture analyzer according to the method reported by *W*. Arisa et al. [57]. A 5 mm diameter cylindrical probe was used to perforate strawberries with two opposite sites along the equatorial plane. The penetration speed was 1 mm/s and the penetration depth was around 8 mm. The maximum force during penetration was recorded as strawberry firmness. 

#### 2.4.2. Determination of pH, Total Soluble Solids, and Titratable Acidity

The pH of the strawberry was determined by using a pH meter. The pH of strawberries was initially measured on the first day before storage and after storage after an interval of time. For each treatment, 3 samples were taken to obtain an average reading. The TSS measurement was obtained using a digital refractometer. The TSSs results were represented as percentages. TA was estimated using the method described by Yan Zhou et al. [58]. The strawberry fruit was mashed and an 8 g sample was added to distilled water and maintained at 60 °C for 30 min on a hot plate. Titration was performed using 0.1 N NaOH, the indicator phenolphthalein, and 10 mL of fruit. The TSS to TA ratio was used to calculate the ripening index by using NaOH.

#### 2.4.3. Determination of Antioxidant Activity and Ascorbic Acid

This assay is based on the measurements of the scavenging ability of antioxidants towards the stable radical DPPH (2,2-diphenyl-1-picrylhydrazyl) reported by R. M.Robles-Sánchez et al. [59]. The strawberries were mashed and squeezed into juice and were added to distilled water. This solution was heated for 30 min. Then, the mixture was run through a UV-VIS spectrophotometer at 517 nm wavelength. Radical scavenging activity was calculated as shown in Equation (2) [60,61]:

(2) $$Antioxidant\;activity\;(\%)=\frac{\left(R_{0}\;–\;R_{1}\right)}{R_{0}}\times 100$$

The ascorbic acid content was determined through the method already presented by Ah-Na et al. [62]. The sample was mashed, and 0.2 g was mixed with 10% metaphosphoric acid. The prepared sample was extracted at 200 rpm for 1 h. After one hour, the extract was filtered with a 0.45 µm syringe filter and analyzed by using HPLC. The ascorbic acid was measured at the wavelength of 254 nm.

#### 2.4.4. Determination of Total Reducing Sugar, Total Phenolic Compounds, and % Infection

TRS was determined using the method reported by Tatjana and Jolanta [63]. Initially, DNS (3,5-dinitrosalicylic acid) solution was prepared by using 0.5 g of DNS in a 50 mL volumetric flask. Then, 20 mL of 2 M NaOH solution was added into the DNS volumetric flask and the flask was filled up to the mark with distilled water. The prepared solution was left at room temperature (27 °C) until it dissolved completely. To measure the TRS, 1 mL prepared DNS solution was added to 5 mL of sample solution in a test tube and the test tube was placed in a hot water bath at 95 °C for 5 min. After 5 min, the absorbance was measured at 540 nm.

The protocol of Anjum et al. [64] was used for the determination of TPC. First, a 1 mg/mL sample was used and mixed using 5 mL Folin–Ciocalteu reagent (FCR) and the prepared sample was diluted with water by 1:10. Then, 4 mL of Na_2_CO_3_ was added to the diluted mixture. The sample was measured using a UV-Vis spectrophotometer at 750 nm. 

The infection percentage was initially assessed visually by observing the fungal growth on the strawberries. It was calculated by selecting 30 strawberries from each treatment using Equation (3) [56].

(3) $$Infection\;(\%)=\frac{Decayed\;fruits}{Total\;fruits}\times 100$$

## 3. Results and Discussion

### 3.1. Fe_3_O_4_ NPs Characterization

The iron precursor used was iron (III) sulfate, since it provides the Fe^3+^ ions that can be easily reduced to form iron oxide. Iron sulfate is convenient because it is commercially available and suitable for green synthesis, in which it very successfully interacts with natural reducers. These ginger and garlic extracts, as eco-friendly reducing agents, are rich in bioactive compounds such as polyphenols and sulfur-containing compounds, which help to reduce Fe^3+^ ions to Fe_3_O_4_ while concurrently stabilizing the nanoparticles. Such extracts also enhance antioxidant and antimicrobial properties, thus fulfilling the Sung criteria of sustainable synthesis without using synthetic chemicals. The lemon juice acts as a natural acidifier to keep the reaction in an adequately controlled acidic state that supports the growth process, increasing the stability parameters of the nanoparticles. Lemon juice provides dispersion of the particles due to the citric acid in it, preventing the aggregation of particles and supporting a uniform size for the nanoparticles in the process. Moreover, the sulfur compounds in garlic and polyphenols in ginger behave like natural capping agents, further stabilizing NPs by attaching to the surface of NPs. This minimizes agglomeration, adding to the biocompatibility. The natural capping layer not only stabilizes the nanoparticles but also enhances their antimicrobial efficiency, which is an added advantage for food preservation purposes. All in all, this comparative analysis underlines how each ingredient provides different types of support for effective green synthesis of Fe_3_O_4_ nanoparticles. Furthermore, the obtained IO NPs were investigated to analyze their physiochemical properties using XRD, FTIR, and SEM for surface analysis.

#### 3.1.1. XRD

**Figure 4:**
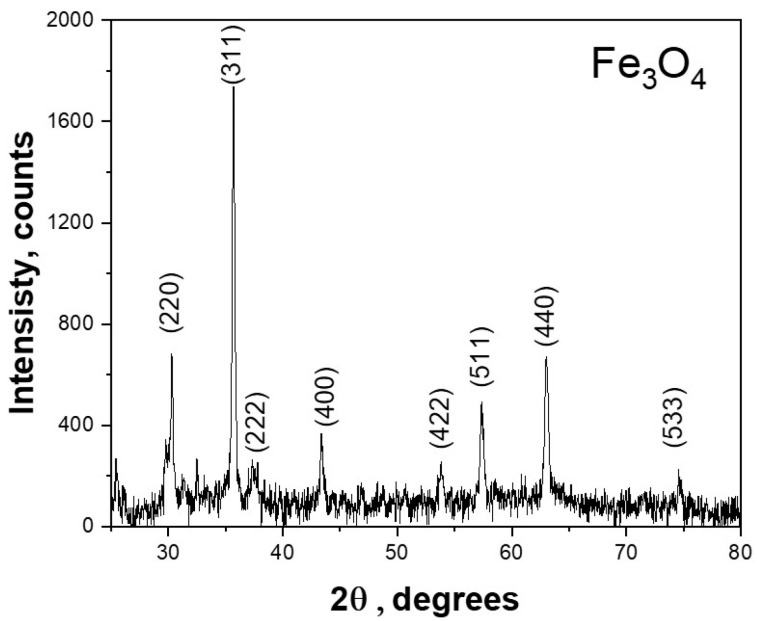
XRD pattern for Fe_3_O_4_ NPs.

XRD patterns showed distinct peaks positioned at 30.2, 35.6, 37.3, 43.2, 53.8, 57.3, 63.1, and 74.6 degrees on the 2θ scale; these findings are in alignment with the Joint Committee on Powder Diffraction Standards (JCPDS) file No. 86-0550 [65]. XRD patterns are shown in Figure 4. These results showed the crystalline constitution of the biosynthesized Fe_3_O_4_. Furthermore, Scherrer’s equation facilitated the determination of the crystalline nature of the Fe_3_O_4_ as well as an average particle size of approximately 48.1 nm, as shown in Table 1.

#### 3.1.2. Scanning Electron Microscopy

**Figure 5:**
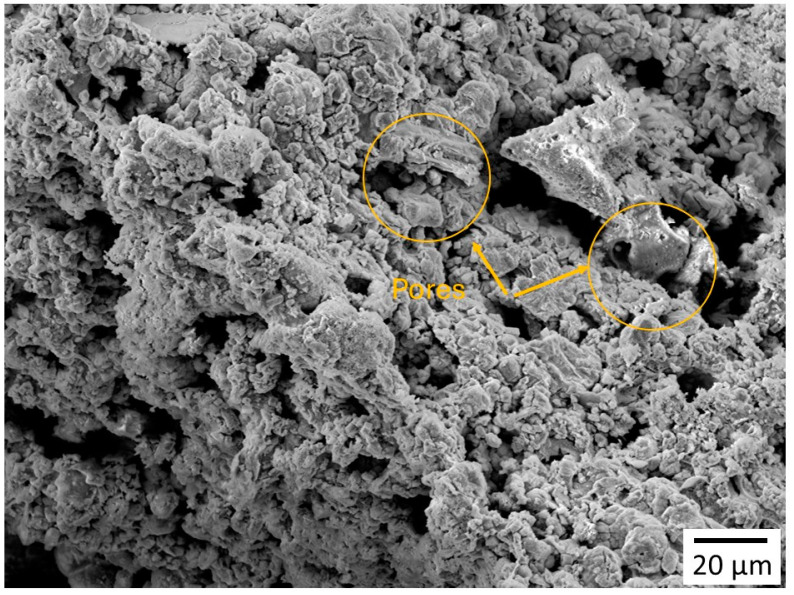
SEM micrograph for Fe_3_O_4_ NP.

The SEM image showed the IO NPs clusters. The clustering of IO NPs could be attributed to their magnetic nature, as maghemite NPs are known for their ferrimagnetic nature. The image further revealed porous structures, which are represented by dark spots as shown in Figure 5. The dark spots represent empty spaces in the clusters. Furthermore, the bright spots represent IO NPs, and the furry structures on the cluster surfaces show that the NPs are not tightly bonded and could be freed up easily, which is a bonus when using these NPs in the CH coating solution.

#### 3.1.3. Fourier Transform Infrared Spectroscopy

**Figure 6:**
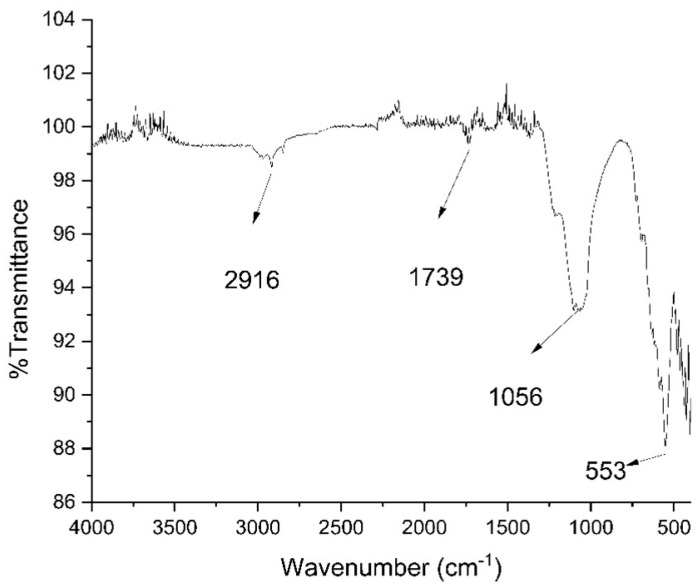
FTIR pattern for Fe_3_O_4_ NP.

The FTIR analysis provided further insights, with peaks appearing at specific wavelengths. One peak appeared at 553 cm^−1^. This peak confirms the synthesis of IO NPs; as reported in the literature, the peak at 553 cm^−1^ represents Fe–O vibrations, as shown in Figure 6. Further peaks at 1056, 1739, and 2916 cm^−1^, represent C–O stretching, C=O stretching, and O–H stretching, respectively. These findings are consistent with the expected chemical structure and interactions within the nanoparticle composite.

### 3.2. Coating Solution and Fruit Characterizations

#### 3.2.1. Scanning Electron Microscopy of Fruit

**Figure 7:**
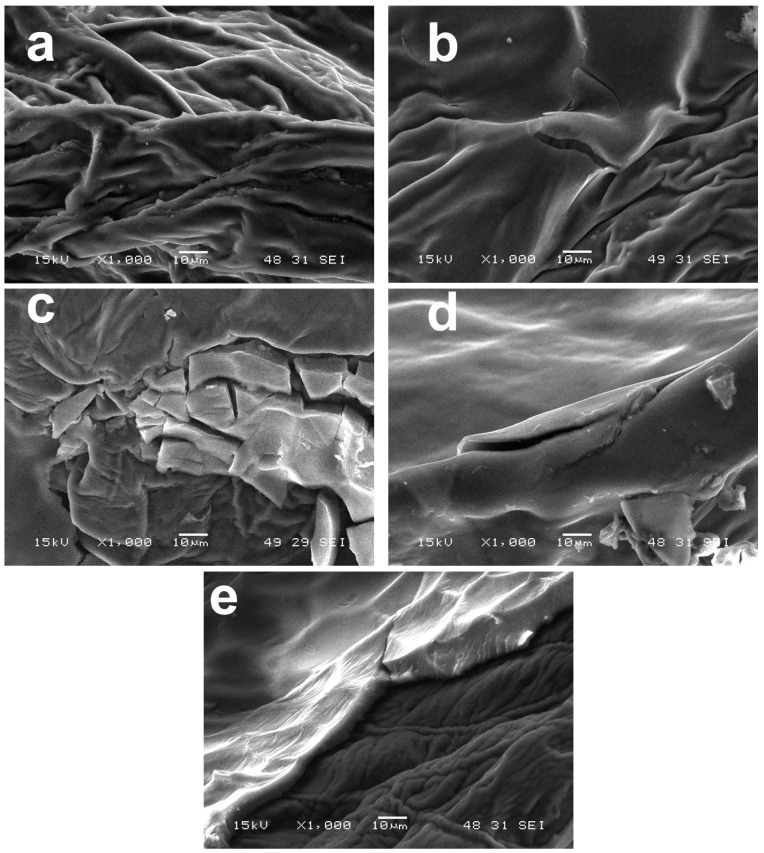
(**a**–**e**) SEM images of strawberry peels.

The SEM examination was conducted to assess the coatings applied to the surface of the strawberries. The images in Figure 7, as documented, show clear distinctions between treated and control samples. The control sample lacks any coating, and therefore the surface roughness is visible, as shown in Figure 7a. Moreover, 1.5% CH coating exhibited a smoother surface, which confirms the appearance of this coating on strawberries. The strawberry samples also included a 1.5% CH coating (Figure 7b) incorporating different concentrations of NPs, including 1% IO, 2% IO and 3% IO NPs. The SEM images clearly show cracked regions within the coatings, confirming the proper application of coating over the surface of strawberries, as shown in Figure 7c–e. The dehydration of the strawberries caused cracks inside the coatings, which are visible in the SEM images. Metal oxide NPs are known to cause ionic bonding [66] as well as hydrogen bonding [67] with the CH structure, limiting the movement of polymeric chains, which creates a compact structure. Furthermore, the NPs also act as a nanofiller [68], occupying the vacant spaces inside the CH structure and improving materials’ mechanical properties, like their hardness, and reducing the flexibility of the coatings. According to the NPs and CH interaction, a higher concentration of NPs would lead to improved mechanical properties, helping to enhance the coated fruits’ shelf-life.

#### 3.2.2. Rheological Properties

**Figure 8:**
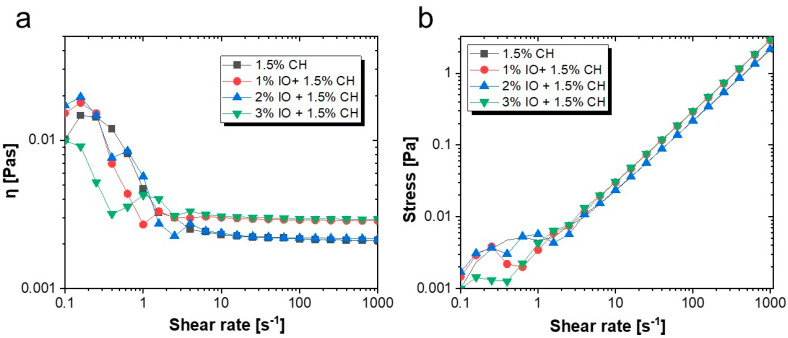
(**a**) Viscosity against shear rate; (**b**) stress vs. shear rate.

Rheology is a crucial aspect of food analysis that provides insights into food materials’ flow and deformation characteristics, especially those of semi-solids or liquids. It plays a vital role in understanding the intermolecular interactions or bonding of food components with other polymers and bio-components. As illustrated in Figure 8a, the viscosity curve is a function of the shear rate. The viscosities are higher at a lower shear rate ranging between 0.1 and 1 due to the resistance of disordered arrangement of IO NPs. Moreover, as the shear rate range becomes higher (>1), it abruptly decreases. This shear-thinning behavior results from the deformation of the internal structure under higher shear rates, leading to the reorientation of particles along the direction of shear. Meanwhile, if the results were compared, the 3% IO NPs concentration exhibited an initial lower increase in viscosity (around 0.01) at a 0.1 shear rate, which continuously decreased until the viscosity stabilized. This behavior suggests that the higher nanoparticle concentration provides a robust chain structure to resist shearing forces [69,70]. We analyzed the flow curve of stress in relation to the shear rate for the coating solution of CH with 1%, 2%, and 3%, IO NPs, and a CH coating without NPs. The results revealed that the shear rate directly relates to stress, as the presence of IO NPs does not affect the flow curve, resulting in similar rheological behavior. This steady behavior declared similar velocities of the material during the applied stress. As shown in Figure 8b, CH without NPs and 2%, 1%, and 3% IO NPs showed an overlap in the shear rate range from 1 to 100 s^−1^, which indicated the stability of synthesized NPs inside the coating solution [71,72].

**Figure 9:**
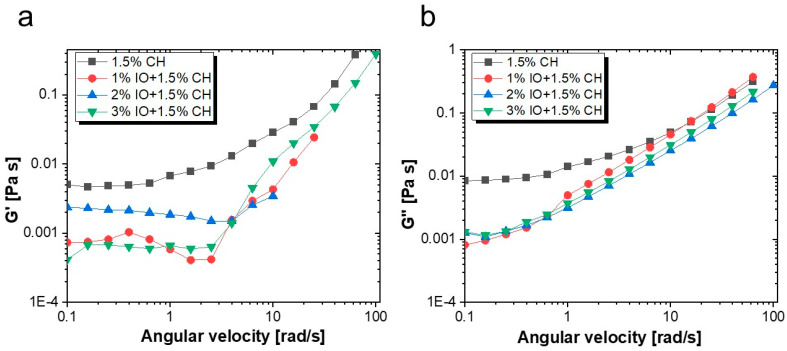
(**a**) Loss modulus; (**b**) storage modulus.

Figure 9 illustrates the G′ and G″ modulus for a 1.5% CH solution and a CH solution containing 1%, 2%, and 3% IO NPs. These solutions contribute to the viscous or elastic material characteristics in an angular frequency range (0.1 to 100 rad/s). The CH solution without NPs and with 1% and 3% IO NPs showed G″ > G′, which revealed dominant viscous behavior. In the case of 2% IO NPs, the G′ > G″ revealed dominant elastic behavior. These concentrations of NPs form chain-like structures and show solid-like behavior as a result [73]. Beyond a specific frequency range, the G′ moduli diminish and the G″ moduli become dominant. Notably, at the end, all results showed dominant G″ (loss moduli) which demonstrated the liquid-like behavior of all the samples. The concentration of NPs was affected by the presence of phytochemicals from the natural extracts. The 3% concentration of IO NPs was considered an optimized concentration in the natural extract-based coating solution, while 1% and 2% concentrations were influenced by interactions between chitosan and phenolic compounds up to a certain threshold. The studies suggest that the coating solution must be applied to strawberries in a load-free environment to allow the coating to form properly. 

### 3.3. Quality Parameters of Food Coatings

Strawberries were stored for 9 days at 27 °C, and their quality parameters were evaluated after a specific time interval.

#### 3.3.1. Determination of Weight Loss and Firmness

**Figure 10:**
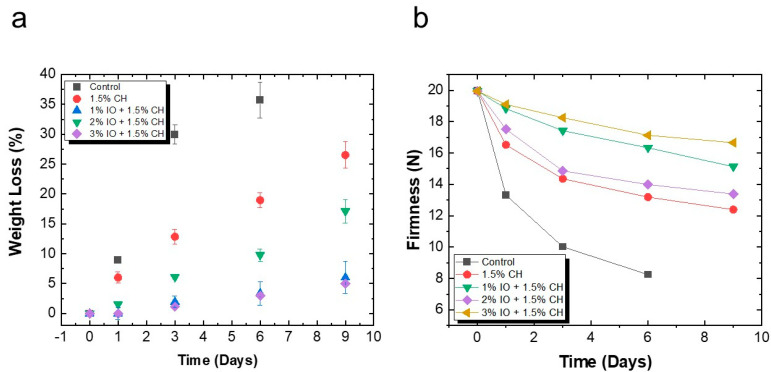
The quality parameters of preserved strawberries at room temperature (27 °C) (**a**) % weight loss (**b**) firmness study. All the values are mean (*n* = 5) ± SD.

The weight loss analysis was conducted to evaluate the impact of storage on strawberries over time. Basically, strawberries have a fragile epidermis layer and exhibit rapid water loss through the pores in their skin, leading to a reduction in the size of the strawberries over time. However, the coated strawberries displayed a smaller reduction in size, as the coating effectively sealed the pores of their skin and lowered the rate of transpiration [74]. The weight loss graphs presented in the referenced figure illustrate the effects of storage at room temperature (27 °C) on weight retention, as shown in Figure 10a. The uncoated control samples experienced a significant weight loss of approximately 35.69% by the sixth day post-harvest. In contrast, the 1.5% CH-coated strawberries devoid of NPs showed a reduction in weight loss of 26.52% on the ninth day of storage. Herein, incorporating IO NPs in different concentrations, including 1%, 2%, and 3%, resulted in weight losses of around 6%, 17%, 4.9%, respectively.

The firmness of the strawberries, an essential quality attribute, was significantly influenced by the application of coating material. The ripening process breaks down the middle lamella in the cell wall, which lowers the strawberries’ capability to withstand load [75]. As shown in Figure 10b, the control sample showed a firmness load of 19.95 N on the day before storage, whereas on the sixth day of storage, it showed a firmness value of 8.25 N. The sample coated with 1.5% CH only tolerated a load of around 12.4 N on the ninth day of storage. Moreover, the CH-coated sample containing IO NPs showed a progressively higher load tolerance on the final day: 1% IO NPs showed load tolerance of 15.13 N, 2% IO NPs showed load tolerance of 13.39 N, and 3% IO NPs showed load tolerance of 16.6 N. This discrepancy is because the coating creates a barrier between the environment and the strawberries, which reduces the amount of cellular damage and causes higher load resistance.

#### 3.3.2. Determination of pH, Total Soluble Solid and Titratable Acidity

**Figure 11:**
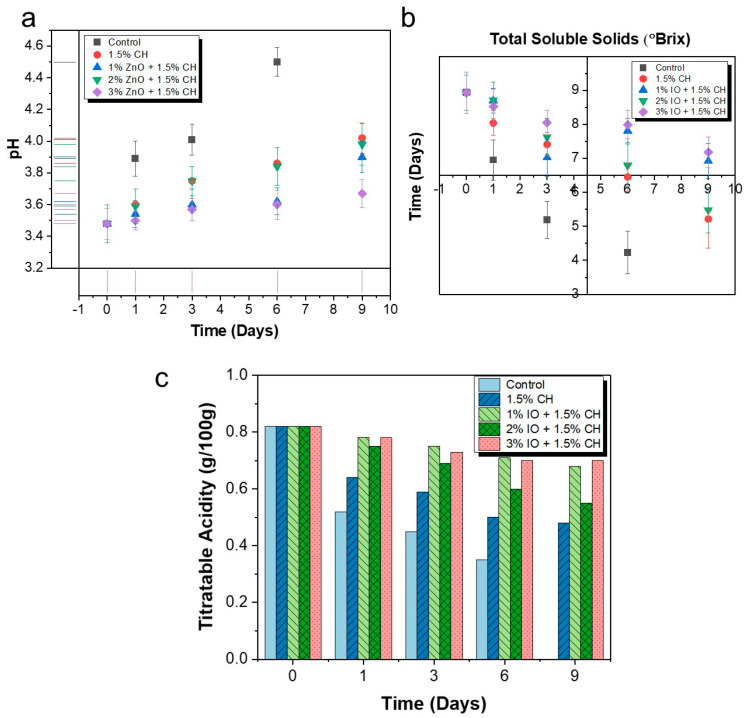
The quality parameters of preserved strawberries at room temperature (27 °C) (**a**) pH; (**b**) TSS; (**c**) TA. All the values are mean (*n* = 5) ± SD.

pH is a fundamental indicator of citric fruits, which shows their ripening and oxidative process. Typically, as citric fruits begin to ripen, their pH value increases, which indicates that the fruit is spoiling. Herein, pH levels were monitored over the storage period to assess the ripening process of strawberries with and without coatings. Initially, the pH values were determined for all the strawberries at around 3.48, considered as day 0. By the end of the storage period, the uncoated strawberry samples had a pH of 4.5, indicating significant ripening. In contrast, the 1.5% CH coating solution had a pH value of approximately 4.02. Afterwards, the CH coating with IO NPs showed significantly less fluctuation, whereas the coating containing 1% IO NPs showed 3.98, that containing 2% IO NPs showed 3.93, and that containing 3% IO NPs showed 3.88 by the ninth day of storage, as shown in Figure 11a. 

TSS serves as a gauge of the flavor of strawberries. TSS is the analysis of fructose, sucrose, glucose, various organic acids, etc. A high sugar content generally indicates a well-balanced flavor [76]. The TSS values show that the smaller the decrease in TSS values, the greater the quality of the fruit. Initially, the TSS value for the strawberries was 8.93. After storage, this value decreased across all samples. The control samples exhibited a TSS value of 4.23 on the seventh day of storage and the CH-only coating showed a TSS value of around 5.21, as shown in Figure 11b. The CH coating with 1%, 2%, and 3% IO NPs showed 5.48, 5.73, and 6.45 at final day of storage, respectively. This observation showed approximately 2.2 °Brix difference in the uncoated and coated samples with 3% IO NPs. 

Strawberry is a citric fruit that contains various organic acids like malic acid and citric acid. A change in fruit acidity basically demonstrates the metabolic activity of the fruit. The measurement of TA is important, as it allows us to understand the biological mechanism which regulates acid and influences the quality of food [77]. The storage was conducted at room temperature, as mentioned in Figure 11c. During storage at room temperature, the uncoated strawberries exhibited a decrease in TA to 0.35, which was indicative of the senescence process in which organic acids are consumed, reducing acidity. In contrast, the CH-coated strawberries retained a higher TA value of 0.48 on the final day post-harvest. The TA value for a 1% concentration was 0.55, the value for a 2% concentration was 0.6, and the value for a 3% concentration was 0.68 on the seventh day of storage. 

#### 3.3.3. Determination of Antioxidant Activity and Ascorbic Acid

**Figure 12:**
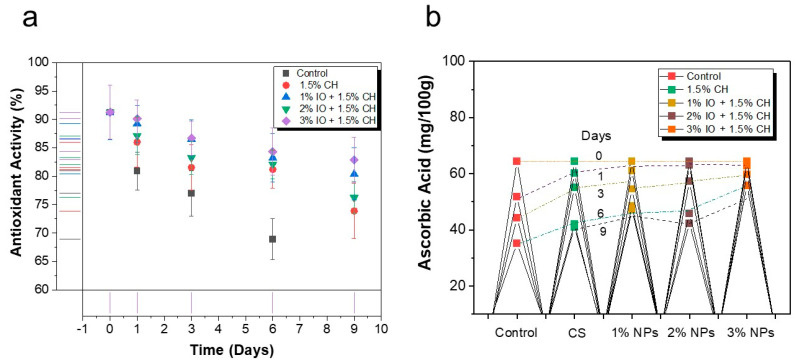
The quality parameters of preserved strawberries at room temperature (27 °C). (**a**) % Antioxidant activity; (**b**) ascorbic acid concentration (%). All the values are mean (*n* = 5) ± SD.

The antioxidant capacity is an important factor used to assess fruits’ quality. The antioxidant activity showed a decline for the uncoated samples due to decay and senescence [78]. The uncoated strawberries displayed antioxidant activity of 70.91% by the seventh day of storage, as shown in Figure 12a. However, strawberries coated with CH showed a slightly higher antioxidant activity of 73.89%. Notably, the addition of IO NPs further enhanced this protective effect. Moreover, the antioxidant activity with IO NPs at a concentration of 1% was 80.37%; for a 2% concentration, it was 76.22%; and for a 3% concentration, it was 82.89% on the final day of storage, respectively. The antioxidant activity shows that the content of the extracts is rich in bioactive compounds, with greater antioxidant properties. Therefore, the coated sample reduces the decay rate as well as maintaining the quality of strawberries [79]. 

Ascorbic acid is considered to be a crucial vitamin in citric fruits such as strawberries. It is a sensitive nutrient compared to other nutrients, and it can easily oxidize during the storage period as the fruit ripens [80]. The results revealed that uncoated samples contained 38.27 mg/100 g ascorbic acid while the CH coating contained 40.98 mg/100 g on the seventh day of storage, as shown in Figure 12b. Conversely, the strawberries, which were coated with coating solution, exhibited enhanced ascorbic acid retention till the final day of storage. A 1% concentration of IO contained 48.28 mg/100 g ascorbic acid, 2% IO contained 42.38 mg/100 g, and 3% IO contained 55.81 mg/100 g, respectively.

#### 3.3.4. Determination of Total Reducing Sugar

**Figure 13:**
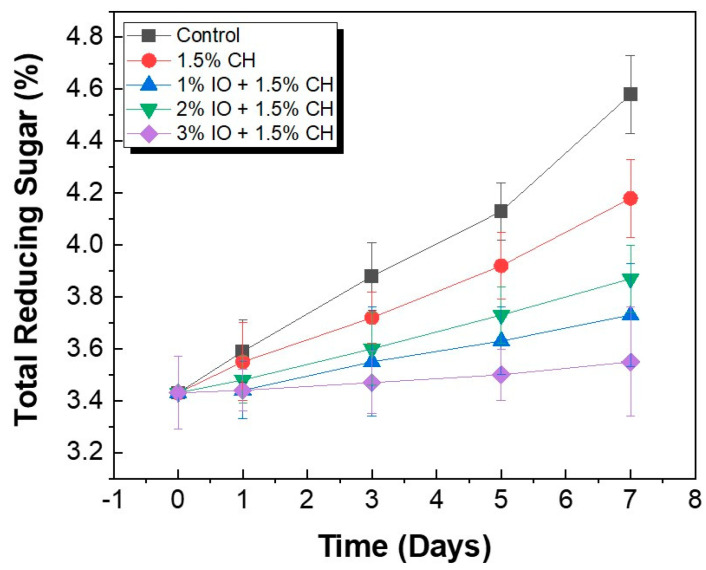
TRS of preserved strawberries at room temperature (27 °C). All the values are mean (*n* = 5) ± SD.

The TRS content is the breakdown of carbohydrates during storage. The study was conducted at room temperature (27 °C). Initially, the TRSs values for all the samples were approximately 3.43% before storage. Uncoated strawberries showed enhanced TRSs values around 4.58% due to the ripening and senescence during storage. The CH coating showed a TRSs value of around 4.18% on the seventh day of storage, as shown in Figure 13. Moreover, 1%, 2%, and 3% IO NPs showed TRSs values of 3.73%, 3.87%, and 3.55%, respectively. As the concentration increased, the TRSs values showed a decline. This trend is due to slowing in decay rate and enhancing shelf life as coating as a metabolic inhibitor.

#### 3.3.5. Determination Total Phenolic Compounds, and % Infection

**Figure 14:**
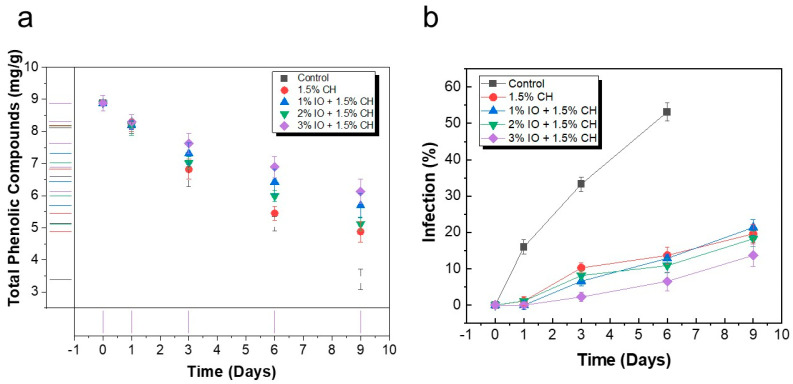
The quality parameters of preserved strawberries at room temperature (27 °C) (**a**) TPC (**b**) % infection. All the values are mean (*n* = 5) ± SD.

Phenolic compounds are considered to be the most important bioactive compounds. The vital phenolic acids present in strawberries are ellagitannins and ellagic acid [81]. Ellagic acid has various biological effects such as anti-inflammatory, antioxidant, and probiotic properties, etc. These acid values fluctuate during the post-harvest stage and during the storage of strawberries, which defines the spoilage of strawberries after a specific amount of time spent in storage [82]. The quantification of TPC stored at room temperature (27 °C) is detailed in Figure 14a. Initially, the TPC values were around 8.8 mg/g. The result indicates that each sample showed a decrease in the TPC value till the final day of storage. The uncoated sample showed TPC values of approximately 3.39 mg/g on the sixth day of storage, whereas the samples that incorporated IO showed TPC values of approximately 5.69 mg/g, 5.1 mg/g, and 6.13 mg/g for 1% IO, 2% IO, and 3% IO concentrations, respectively. The reduction in the TPC concentration in 3% IO NPs-coated strawberries was 1.43 times smaller than that of the uncoated control strawberries due to the improved barrier properties.

The percentage of infection was determined throughout the storage period. The control sample showed 53.13% infection on the seventh day of storage because strawberries have fragile and delicate skin, causing them to lose moisture content and increasing their chances of infection. Moreover, the coating solution made of CH in conjunction with ginger–garlic extract extracts and incorporating IO NPs acted as a barrier for the strawberry samples. Therefore, the coated sample showed less mold growth than the uncoated samples. The 1.5% CH coating showed a % infection of approximately 19.65%, whereas the 1% IO coating showed 18.25% infection, 2% IO showed 21.36% infection, and 3% IO showed 13.68% infection on the final day of storage, as detailed in Figure 14b.

## 4. Conclusions

The current research was conducted on natural extracts of CH-based coatings with various concentrations of Fe_3_O_4_ NPs. Natural extracts were used for coating preparation, including ginger and garlic extract and lemon juice, to enhance the chemical properties. The integration of CH with iron oxide nanoparticles, alongside natural extracts, significantly boosted the antioxidant and antimicrobial properties of the coatings. The presence of IO NPs in the CH polymeric matrix facilitated the production of a higher number of reactive oxygen species (ROS) on the surface of the strawberries, complementing the intrinsic antimicrobial properties of the CH [83]. This synergistic combination effectively slowed the ripening process and extended the shelf-life of the treated produce. The results of the investigation demonstrated marked improvements across all assessed quality parameters. The weight loss and infection study showed approximately 21.6% and 6% decreases for 3% IO with 1.5% CH coating solution compared to 1.5% CH coating solution, respectively. Moreover, the TSSs, TRSs, and TPCs for 1.5% CH that incorporated 3% IO NPs were around 6.45 °Brix, 3.55%, and 6.13 mg/g, respectively. These findings underscore the effectiveness of the nanoparticle-enhanced coating in not only preserving the structural integrity and quality of the strawberries but also extending their shelf-life with minimal impact on their quality during storage. This research presents significant insights and lays the groundwork for future studies, highlighting the potential of CH-IO NP coatings as a promising approach for food preservation. The promising results invite further exploration into similar coatings, potentially using CH combined with IO NPs to broaden the scope and application of this technology in food science.

## 5. Future Directions

In this study, we have successfully developed Fe_3_O_4_ NPs-containing CH coatings synthesized by employing all-natural resources, including lemon juice, ginger, and garlic extracts. The coatings showed promising results and safeguarded the tested strawberries during storage. It is recommended to study lab-based and animal-related toxicity studies of the Fe_3_O_4_ NPs-containing coatings in the future. Although Fe_3_O_4_ has FDA approval, a range of toxicity evaluation studies are still recommended.

## 6. Project Details

This research, which forms part of a PhD thesis, introduces a novel method for developing a CH coating utilizing naturally sourced extracts and juices. It further investigates the effects of various NPs—such as ZnO, Fe_3_O_4,_ NiO—on the chemical properties of coatings on the shelf life of strawberries. Detailed reports on these impacts will be published separately to fulfill the PhD thesis requirements.

## Figures and Tables

**Table 1:** Calculated crystalline size of Fe_3_O_4_ NP.

Peak	Size, nm
220	75.56
311	32.27
222	78.82
400	42.92
422	30.81
511	40.33
440	42.90
533	41.50
**Average Crystalline Size**	**48.138**

## Data Availability

The original contributions presented in the study are included in the article, further inquiries can be directed to the corresponding author.
